# The Use of Dog Collars Offers Significant Benefits to Rabies Vaccination Campaigns: The Case of Zanzibar, Tanzania

**DOI:** 10.3390/tropicalmed8080421

**Published:** 2023-08-21

**Authors:** Khadija N. Omar, Andre Coetzer, Maulid Hamdu, Ayla J. Malan, Ali Z. Moh’d, Talib S. Suleiman, Louis H. Nel

**Affiliations:** 1 Zanzibar Livestock Research Institute, Zanzibar P.O. Box 104, Tanzania; 2 Global Alliance for Rabies Control South Africa Non-Profit Company, Pretoria 0181, South Africa; 3 Department of Biochemistry, Genetics and Microbiology, Faculty of Natural and Agricultural Sciences, University of Pretoria, Pretoria 0002, South Africa; 4 Department of Livestock Development, Ministry of Agriculture, Irrigation, Natural Resources and Livestock, Zanzibar P.O. Box 159, Tanzania

**Keywords:** rabies, dog, collar, survey, KAP

## Abstract

Tools and resources that could increase dog vaccination coverage have become increasingly critical towards progressing the goal to eliminate dog-mediated human rabies by 2030. In this regard, dog collars that are fitted during vaccination campaigns could potentially enhance owner participation. The use of dog collars will, however, increase the cost per dog vaccinated and the impact and benefit of this practice should be elucidated. This study evaluated the impact of dog collars by testing the perception and related behavioural influences in communities in Zanzibar. In this cross-sectional investigation—conducted approximately two months after the implementation of a mass dog vaccination (MDV) where dog collars were provided to vaccinated dogs—data were collected from 600 respondents in 56 municipal wards in Zanzibar. Descriptive analyses and logistic regressions were undertaken to determine the impact the collars had on respondents with regards to (i) engaging with the community dogs, (ii) health seeking behaviour after exposure, and (iii) overall participation during dog vaccination campaigns. From the data, it was evident that the collars had a positive impact on the community’s perception of dogs, with 57% of the respondents feeling safer around a dog with a collar, while 66% of the respondents felt less safe around a dog without a collar. Furthermore, the collars had a positive impact on participation during dog vaccination campaigns. Of the 142 respondents who owned dogs, 64% reported that the collars made them more likely to take their dogs for vaccination, and 95% felt that the collar was an important sign of the dog’s vaccination status. This study demonstrated that dog collars could not only improve participation during dog vaccination campaigns, but that they could also play a significant role in the community’s perception of rabies vaccination campaigns and vaccinated dogs in general.

## 1. Introduction

Rabies is vaccine preventable, yet continues to afflict tens of thousands of people every year—primarily children and resource-limited families in low- and middle-income countries. Based on modelled disease burden data, rabies deaths in humans are thought to exceed 59,000 deaths per year, while the rabies-related death toll in animals is considerably higher. Africa and Asia are most widely affected by rabies, with the domestic dog (*Canis lupus familiaris*) being the principal reservoir, and responsible for the majority of the human rabies cases reported annually [1]. As the only vaccine-preventable neglected tropical disease, the routine vaccination of dogs has been shown to be effective in the control and elimination of dog rabies and dog-transmitted human rabies [2,3,4,5]. This approach is, however, reliant on the vaccination of a significant proportion of the vulnerable dog populations in order to be effective [2,3,4,5]. While reaching the recommended vaccination coverage of 70% is known to be effective, mass dog vaccination (MDV) campaigns are typically marred by a lack of resources, limited inter-sectorial collaboration, and limited availability of vaccines—with the resulting number of dogs vaccinated often being too few to make any noteworthy or sustained difference to the incidence of the disease [1,2,5,6,7]. In addition, the success of MDV campaigns are also reliant on community participation, which requires effective communication, advocacy and awareness in the target communities. However, even with the aid of advocacy and awareness messages, studies have reported instances where dog owners were unwilling to take their dogs to the vaccination sites, resulting in reduced vaccination coverage despite the availability of capacity and resources [7,8,9].

One aspect that could be further explored is whether dog collars could make a positive contribution towards MDV campaigns in terms of community attitude and participation. In the past, dog collars have in some studies been used as identifiable markings that could be used to estimate dog population sizes [10,11,12,13,14], determine the number of vaccinated dogs, and calculate the subsequent vaccination coverage by means of mark-resight studies [15,16,17,18,19,20,21]. Those studies mostly considered the practical benefit of dog collars to estimate vaccination coverage. In fact, very few published studies that we are aware of considered the potential impact of dog collars on the general perception of community dogs—especially those wearing collars as an indicator of their rabies vaccination status.

One study, aimed at agro-pastoralist communities of two districts of the Mara region in northwestern Tanzania, showed that incentives such as dog collars and wristbands for owners increased owner participation across 10 fixed point vaccination clinics carried out in December 2013. In that study, the authors noted that an average of 34 more dogs were vaccinated in villages where incentives were handed out, while also noting that the incentives failed to increase the vaccination coverage above the required threshold of 70% [7].

The second notable study reported on the ‘Red Collar’ campaign, which was implemented between 2011 and 2016 by the World Animal Protection (WAP)—formerly known as the World Society for the Protection of Animals—in collaboration with the governments of Bangladesh, China, Indonesia, the Philippines, and Zanzibar [22]. The overall objective of that campaign was to promote the use of dog collars to indicate that dogs had been vaccinated against rabies to end the inhumane culling of dogs in response to rabies. At the end of the five-year campaign, relying on the distribution of key advocacy messages and pilot vaccination campaigns in the target countries, a qualitative study highlighted key successes and shortcomings associated with the campaign [22]. Firstly, the campaign had succeeded in changing the policy with regards to humane rabies control (*viz*. dog vaccination and not inhumane culling) at either the central or national level in the target countries. Despite changes in policy, the implementation thereof at the local-level was found to remain challenging in most of the countries, with the exception of Zanzibar, where humane rabies control was widely practised. It was noted that the government of Zanzibar’s relationship with WAP exceeded the five years of the ‘Red Collar’ campaign, which could explain the widespread adoption and implementation of the humane rabies control policy [22]. Secondly, the inhumane culling of dogs in the Philippines and Zanzibar decreased, while a two-pronged approach of rabies vaccination and inhumane culling was implemented in other target countries. It was suggested that the decrease in inhumane culling in the Philippines and Zanzibar could not solely be attributed to this campaign, as both countries had been subjected to targeted awareness campaigns focussed on dog welfare and the inhumane nature of culling by WAP and other animal welfare organizations. In addition, the potential impact WAP’s long-standing collaborative relationship with the government of Zanzibar had on the reduced dog culling rates was highlighted [22]. While the ‘Red Collar’ campaign had attained seemingly significant achievements in terms of the creation and implementation of policies related to humane rabies control, the campaign did not benefit from robust data collection and analysis [22]. As such, it did not generate sufficient objective data against which its effectiveness could be measured. 

It is clear that dog collars serve a practical role during and after rabies vaccination campaigns (e.g., mark re-sight studies), while evidence suggests that collars could provide a benefit to the individual dogs wearing the collars (e.g., indicating their vaccination status). To the best of our knowledge, however, there have been no in-depth studies on how the behaviour of community members changes if roaming dogs wear collars that specifically indicate that they have been vaccinated against rabies. The aim of this investigation was thus to address this shortcoming through the implementation of a knowledge, attitude, and practice (KAP) survey using a cross-sectional questionnaire after the implementation of a MDV campaign using bright yellow dog collars as markings in Zanzibar. The implementation of this study in Zanzibar—which has a rich history of both large-scale MDV campaigns [23] and dog collar usage [22]—provided key findings in terms of the community’s behaviour with regards to (i) engaging with community dogs, (ii) health seeking behaviour after exposure, and (iii) participation in rabies vaccination events.

## 2. Materials and Methods

### 2.1. Dog Vaccination and the Use of the ‘Rabies Vaccination’ Collars

**Figure 1:**
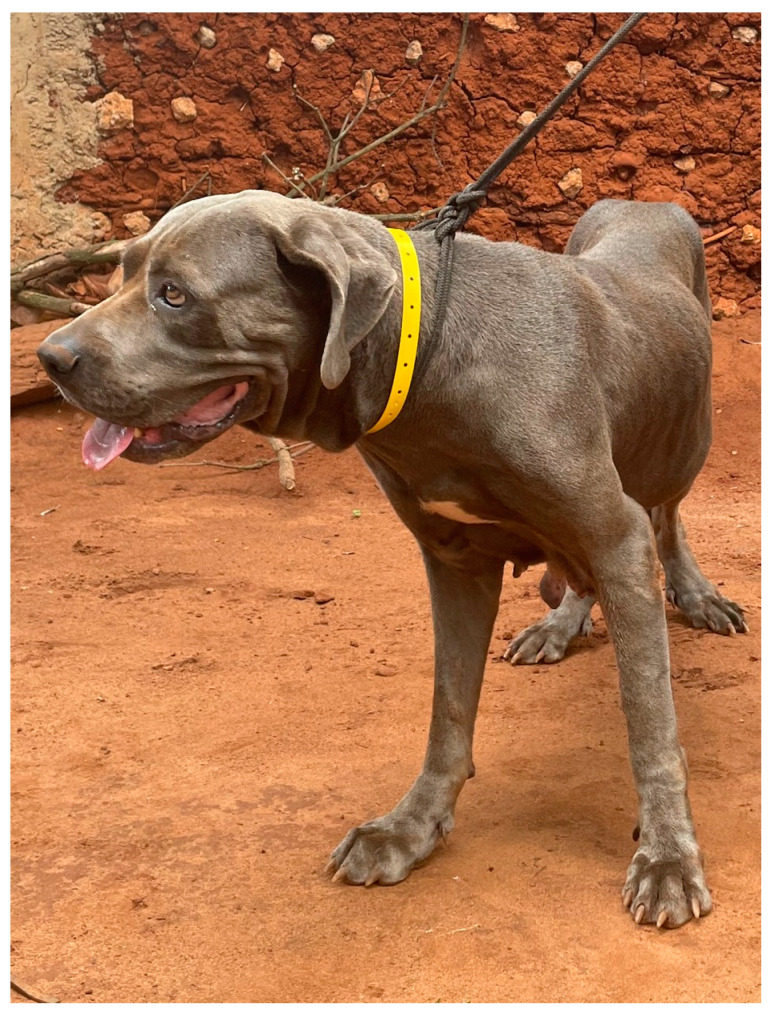
A vaccinated dog fitted with the yellow collar.

Between 28 November and 31 December 2022, 4100 dogs were vaccinated against rabies across the island of Zanzibar by district veterinary officers (DVOs)—with the location and time of each vaccination captured using a mobile phone application [23,24]. In addition, each vaccinated dog was fitted with a bright yellow collar (Figure 1). After placing the collars on the dogs, dog owners and community leaders were informed that the collars distinguished these dogs as having been vaccinated against rabies and owners were requested to leave the collars on the dogs for as long as possible. No other supporting information or awareness materials were given to owners whose dogs had been vaccinated and fitted with a collar during the campaign. 

### 2.2. Study Area for Survey

**Figure 2:**
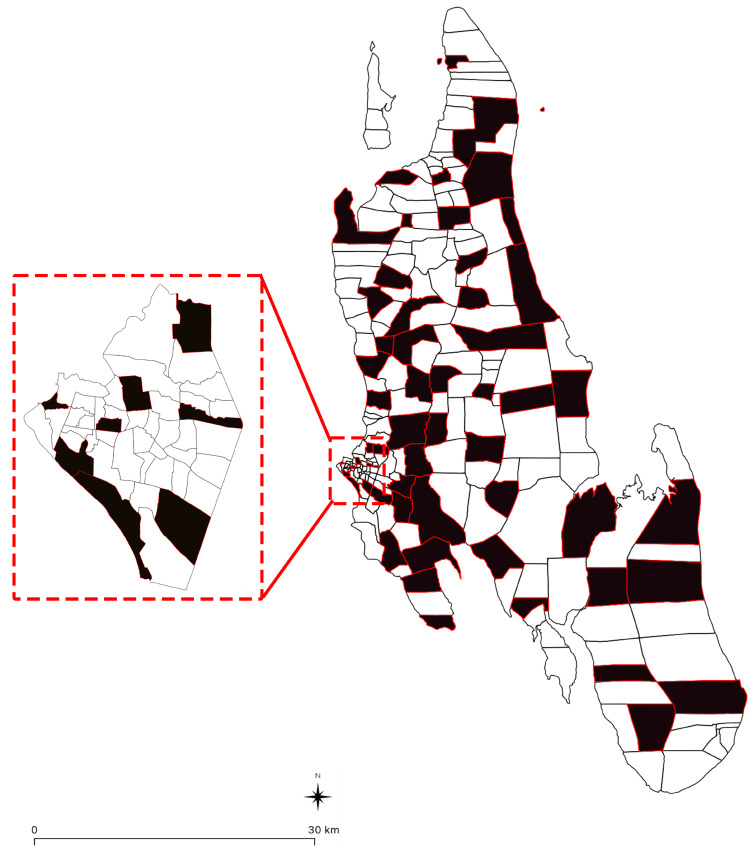
Map of Zanzibar showing locations of sampled wards (shown in black).

The survey was conducted in 56 municipal wards across the island of Zanzibar, Tanzania (Figure 2). The wards were selected based on where dogs had been vaccinated during the 2022 vaccination campaign, as well as the geographical distribution of the wards across the island—aiming to include wards in both urban (areas with high population density and primarily built-up infrastructure) and rural (areas with a lower population density and primarily limited infrastructure) districts in the study (Figure 2).

### 2.3. Study Design and Sample Size

As no similar studies had previously been conducted in Zanzibar, we assumed that 50% (default) of the respondent would have knowledge and awareness about the dog collars. Allowing a margin of error (confidence interval) of 4% and a 95% level of confidence, the minimum sample size (*n* = 400) for this study was determined using a website-based sample size calculator (https://www.surveysystem.com/sscalc.htm, accessed on 6 December 2022). The semi-structured questionnaire (Supplementary File S1) was pre-tested in one ward (*n* = 10 respondents) on the first day (1 February 2023) and no shortcomings to the questionnaire were identified. The questionnaire was subsequently implemented across the remaining 55 wards between 2 and 16 February 2023 by seven teams, with each consisting of a DVO and a researcher from the Zanzibar Livestock Research Institute (ZALIRI) (Supplementary File S2).

As part of a pre-survey workshop, the surveyors were given in-depth training on the correct interpretation questionnaire (Supplementary File S1) and given instructions that the respondents had to be selected randomly with the only exclusion criteria being their age, as the respondents in the study had to be 18 years or older.

Throughout the survey, surveyors traversed the streets and randomly approached both male and female community members that were outside of their households at various points in the ward. During the face-to-face interviews, the respondents entered into a consent dialogue with the surveyors. To do this, the surveyors explained the purpose of the study without specific mention of the dog collars. Thereafter, all respondents were informed that the survey was anonymous and that they could voluntarily withdraw without penalty at any point during the interview. Respondents—who were all 18 years and older and had freely given their verbal consent to participate in the study—were interviewed in Swahili and the answers were captured using an online questionnaire developed using Google Forms. Individuals who did not wish to participate, and thus did not give verbal consent, were omitted from the study and not interviewed. 

### 2.4. Statistical Analysis

Data collected from each questionnaire were captured in Microsoft Excel, filtered, and checked for completeness before a descriptive analysis was conducted for the entire dataset. After the socio-demographic characteristics had been summarised, the general perception of people towards community dogs and dog owner behaviour during rabies vaccination events were examined using logistic regression modelling. As a first step, a univariable logistic regression analysis was used to calculate the odds ratios (ORs) and corresponding 95% confidence intervals (CI) for the captured socio-demographic characteristics. Thereafter, a forward stepwise multivariate logistic regression analysis was undertaken as described elsewhere [25,26]. To assess confounding effects, predictor variables that were not selected for the final logistic regression model were added and their impact on the coefficient of the predictor variables determined as described elsewhere [25,26]. No such confounders were detected in this study and the adjusted OR (aOR) and their corresponding 95% CIs were derived.

## 3. Results

### 3.1. Socio-Demographic Characteristics

The socio-demographic characteristics of the 600 respondents included in the study are summarized below. Of the 600 respondents, 75% were male and 25% were female, with a mean age of 37 years (min: 18 years and max: 92 years). Of the 600 respondents, 37% (221/600) were from primarily urban districts (Urban, West A, and West B districts) and 63% (379/600) were from primarily rural districts (Central, South, North A, and North B). Lastly, 29% (175/600) of the respondents were dog owners (Table 1).

### 3.2. General Perception of Community Dogs

Of all respondents, 44% (262/600) had seen dogs wearing the collars in their communities. Of those respondents who had seen the dog collars, 78% (205/262) knew that the collars meant that the dog had been vaccinated against rabies specifically, while 13% (33/262) knew the dog had received some sort of injection before receiving the collar. With regards to the perception of community dogs, 57% (341/600) of all respondents said that they felt safer when encountering a dog with a collar, and 50% (300/600) reported they would act indifferently (e.g., any indifferent action such as ignoring them) towards those dogs in the community. In contrast, 66% (398/600) of all respondents said that they felt less safe when encountering a dog without a collar, and 56% (334/600) reported that they would actively try to stay away from those dogs in the community. Lastly, of the 96 respondents who had observed dogs being killed in the community, 16% (15/96) reported dogs with collars being killed and 70% (67/96) reported dogs without collars being killed (Table 2).

A multivariable logistic regression analysis demonstrated that ‘feeling safer when encountering a dog wearing a collar’ was statistically associated with the predictor variables of ‘setting’ and ‘dog ownership status’ (Supplementary File S3, Table S1). Respondents who owned a dog(s) had 8.12 greater odds of feeling safer when encountering a dog with a collar compared with respondents who did not own a dog (aOR: 8.12, 95% CI: 4.99–13.24). In addition, respondents who lived in a rural district had 2.84 greater odds of feeling safer when encountering a dog with a collar compared with respondents who lived in an urban district (aOR: 2.84, 95% CI: 1.94–4.14) (Supplementary File S3, Table S1).

Furthermore, the multivariable logistic regression analysis demonstrated that ‘feeling less safe when encountering a dog without a collar’ was also statistically associated with the predictor variables of ‘setting’ and ‘dog ownership status’ (Supplementary File S3, Table S2). Respondents who owned a dog(s) had 3.18 greater odds of feeling less safe when encountering a dog without a collar compared with respondents who did not own a dog (aOR: 3.18, 95% CI: 1.65–3.37). Respondents who lived in a rural district had 2.36 greater odds of feeling less safe when encountering a dog without a collar compared with respondents who lived in an urban district (aOR: 2.36, 95% CI: 2.04–4.49) (Supplementary File S3, Table S2).

### 3.3. Health-Seeking Behaviour

Of the 600 respondents, 1% (8/600) had been bitten by a dog in the four weeks leading up to the survey. Of those, 75% (6/8) sought treatment after exposure, with ‘dog bite treatment’ being the most reported reason for seeking primary healthcare (4/6, 67%). Neither of the respondents who did not seek treatment (2/8) were influenced by the presence of dog collars when deciding to forego treatment at a healthcare facility (Supplementary File S3, Table S3).

### 3.4. Dog Owner Participation during Rabies Vaccination Events

Respondents who owned dogs (175/600) had a mean of three dogs per household (range: 1–15) with 70% (122/175) of the respondents reporting that all their dogs had been vaccinated during the previous campaign. Of those respondents who had at least one of their dogs vaccinated during the previous vaccination campaign, 93% (142/152) had received collars for their dogs. Of those, 64% (91/142) reported that receiving the dog collars made them more likely to take their dog(s) for rabies vaccination and 95% (135/142) believed that receiving a collar was an important sign that the dog had been vaccinated against rabies specifically (Table 3).

Despite these observations, 57% (81/142) of the respondents whose dogs had received collars reported that some of their dogs had lost their collars and 10% (14/142) reported that none of their dogs still had their collars on at the time of survey. Of the respondents who had dogs that lost their collars, 9% (9/95) of the dogs wore the collar less than a week, 7% (7/95) of the dogs wore the collar for one week, 22% (21/95) of the dogs wore the collar for two weeks, 25% (24/95) wore the collar for three weeks, 14% (13/95) wore the collar for four weeks, and 22% (21/95) of the dogs wore the collar for more than four weeks. ‘Owners’ and ‘other dogs’ were equally to blame for the lost collars, with 17% (16/95) of the respondents reporting that they removed the collars themselves and 15% (14/95) of the respondents reporting that the dogs lost their collars while hunting or playing/fighting with other dogs (Table 3).

The multivariable logistic regression analysis demonstrated that the variable ‘setting’ was strongly associated with dog owners being more likely to take dogs for vaccination if dog collars were given (Supplementary File S3, Table S4). Compared with respondents who lived in urban districts, respondents who lived in rural districts had a 9.24 lower odds of being more likely to take dogs for vaccination if collars were given to the dogs (aOR: 0.11, 95% CI: 0.03–0.38) (Supplementary File S3, Table S4).

## 4. Discussion

With the ‘Global Strategic Plan to end human deaths from dog-mediated rabies’ (Zero by 30) relying on a country-centric approach [27], it is vital that governmental stakeholders utilize any other tools and resources [24,28,29] at their disposal during MDV events. The use of dog collars is one such resource to improve compliance at the community level. While this would seemingly make dog collars a valuable addition to any vaccination campaign, their use would contribute towards an increase in the overall cost and time required to vaccinate each dog, and the additional value of using the dog collars should be carefully considered. Taking this into consideration, this study endeavoured to determine whether using collars provided any additional benefit to people sharing their communities with the vaccinated dogs in Zanzibar. 

The results of this study suggest that the use of the dog collars had an overall positive impact on the community’s general perception of dogs. For example, 57% of the 600 respondents reported that that they felt safer when encountering a dog with a collar, while 66% of the 600 respondents reported that they felt less safe when encountering a dog not wearing a collar. While the presence of the collars impacted how the respondents felt about the dogs in general, they had no noteworthy impact on whether the respondents would be more inclined to physically engage with the dogs. Considering that approximately 98% of Zanzibar’s population is of the Muslim faith [30]—which considers dogs to be impure animals with saliva that voids a Muslim’s ritual purity [31]—it was not surprising that few respondents were willing to interact with dogs, regardless of the presence of a collar or not. However, it was interesting to note that 58% of the 600 respondents felt indifferent towards dogs with collars compared with 56% of the 600 respondents who reported that they would actively try to stay away from dogs without collars. This would suggest that dogs not wearing the collars were more likely to be ostracized from the community because they had no visible indication of their vaccination status. 

In addition to gaining insight into the community’s general perception of vaccinated dogs, the impact of the collars on dogs being killed by community members was also investigated. The results of our study suggest that dogs without collars were four times more likely to be killed by community members compared with dogs wearing collars. The specific reasons the dogs without collars were killed were higher for all the suppositious options listed in the questionnaire (Table 2). Of particular interest, fewer dogs with collars were killed for ‘biting someone’ or ‘being a nuisance’ specifically (7% with collars vs. 22% without collars for both reasons) (Table 2). While any endeavour that results in fewer dogs being killed for being a nuisance should be considered a positive outcome in terms of animal welfare, fewer dogs being killed for simply biting someone was considered a noteworthy behavioural change, as dog bites could be caused by various factors not related to rabies, *viz*. fear, resource and territory protection, response to pain, protection of puppies, and predatory behaviour [32,33,34]. 

The collars had no influence on health seeking behaviour—but only 1% (8/600) of the respondents had been exposed to a dog bite in a four-week period leading up to the survey, with six of them seeking dog bite treatment (Supplementary File S3, Table S3). The ongoing need to seek primary healthcare was not considered a negative outcome as not all dog bites are from rabid dogs that require rabies post-exposure prophylaxis (PEP) [1,35,36,37], but primary wound treatment is still required as all dog bites are associated with a high risk of infection that could be prevented through wound washing, the use of antibiotics, and the administration of a tetanus vaccine if required [38].

Lastly, the outcomes of this study suggest that the use of the collars had a positive impact on the community’s participation during dog vaccination events. More specifically, 64% of the respondents that had at least one of their dogs vaccinated reported that receiving the collars made them more likely to take their dog(s) for rabies vaccination during future vaccination campaigns (Table 3). The positive impact on owner participation during MDV campaigns was also observed in one other published study that found that incentives such as dog collars increased owner participation during vaccination events in the Mara Region of Tanzania [7]. Our study, however, found that the collars were not only seen as a gift to entice the dog owners to attend dog vaccination events, but that 95% of the respondents that had at least one of their dogs vaccinated felt that the collar was an important sign that indicated the dog’s vaccination status specifically (Table 3).

While the results of our study suggested that the collars contributed towards both the community’s perception of dogs and owner participation during MDV campaigns, the loss of collars was also an important factor to consider, as this would impact the duration of the benefits and findings discussed here. From the data presented here, 67% of the respondents whose dogs had received collars had reported that the collars had been lost by some (or all) of their dogs by the time the survey was conducted (Table 3). The loss of dog collars after rabies vaccination events was also observed elsewhere in the Bali province of Indonesia [19], the Sorsogon province of the Philippines (10% of the dogs had lost their collars after 5 days) [20], and the Iringa region of Tanzania (14% of the dogs had lost their collars after 3 days) [15]. Our study, however, found that 90% of the respondents who had dogs that had received collars reported that some (or all) of their dogs still had the collars on at the time of the survey (average of 62 days between the MDV campaign and the survey (Min: 43 days, Max: 76 days)) (File S2) and that 36% of the dogs that did lose their collars wore them for more than 4 weeks (Table 3).

Our study did have some limitations that should be mentioned. While the ‘Red Collar’ campaign—implemented in Zanzibar between 2011 and 2016—failed to report any concrete findings on the use of the dog collars [22], and the institutional knowledge imparted by the campaign on the dog owners of Zanzibar could not be ruled out. Any significant bias is, however, unlikely, given that at least seven years elapsed since the ‘Red Collar’ campaign. Should any residual knowledge from that campaign have persisted, it would, in fact, support the endearing value of collaring as a component of MDV and it might have been useful to test this element as well.

Despite applying a random sampling method during the survey, responses from a disproportionate number of male respondents formed part of the results of the study. While this could have been due to sampling bias during the survey, we cannot rule out the unwillingness of female members of the public to participate in the study as no information was captured for people that were not willing to participate. It is thus plausible that females were not willing to participate in the study, but instead recommended that the male head of the household respond to the questionnaire.

Lastly, the sample size of respondents who had sought healthcare in response to dog bites was insufficient and no noteworthy statistical analyses of their health-seeking behaviour could be undertaken in this study specifically.

## 5. Conclusions

This study demonstrated that the use of collars to distinguish rabies vaccinated and unvaccinated dogs could not only improve the participation of owners during MDV campaigns, but also play a significant positive role in the community’s perception of rabies vaccination campaigns and vaccinated dogs in general.

We also found that the durability of the dog collars is an important factor and further development in this regard would continue to add value. Inexpensive, yet durable collars that are safe, not easily removed (accidentally), and valued by dog owners would amplify their usefulness to campaigns. Given these qualifications, it is our conclusion that dog collars would benefit most (if not all) dog vaccination events and could be helpful in progressing the global ‘Zero by 30′ aspirations.

## Figures and Tables

**Table 1:** Socio-demographic characteristics of the respondents included in the study.

Characteristic	Frequency *n* (%)
**Sex**	
Female	151 (25.17)
Male	449 (74.83)
**Age**	
18–29	221 (36.83)
30–39	161 (26.83)
40–49	100 (16.67)
50–60	71 (11.83)
>60	47 (7.83)
**Setting**	
Urban	221 (36.83)
Rural	379 (63.17)
**Dog ownership**	
Yes	175 (29.17)
No	425 (70.83)

**Table 2:** General perception of community dogs in the study area.

Characteristic	Frequency *n* (%)
**Types of dog collars observed by respondents in the community (respondents, *n* =600)**	
Rabies vaccination collars	262 (43.67)
Any other dog collar	92 (15.33)
No collars observed	246 (41.00)
**Reason for dogs wearing the collars (respondents, *n* = 262)**	
The dog is vaccinated against rabies	205 (78.24)
The dog has been given an injection (rabies vaccination not mentioned specifically)	33 (12.60)
The dog is healthy	2 (0.76)
The dog has a specific owner	16 (6.11)
Other	6 (2.29)
**Respondent’s perception of dogs wearing collars in the community (respondents, *n* = 600)**	
Feeling safer	341 (56.83)
No change in perception	157 (26.17)
Feeling less safe	102 (17.00)
**Respondent’s reaction towards dogs wearing collars in the community (respondents, *n* = 600)**	
Try to stay away from it	154 (25.67)
Try to chase it away (e.g., throwing something at it or trying to hit it with something)	41 (6.83)
Interact with it (e.g., touching, petting, etc.)	105 (17.50)
No reaction (e.g., any indifferent action like ignoring them)	300 (50.00)
**Respondent’s perception of dogs not wearing collars in the community (respondents, *n* = 600)**	
Feeling safer	46 (7.67)
No change in perception	156 (26.00)
Feeling less safe	398 (66.33)
**Respondent’s reaction towards dogs not wearing collars in the community (respondents, *n* = 600)**	
Try to stay away from it	334 (55.67)
Try to chase it away (e.g., throwing something at it or trying to hit it with something)	99 (16.50)
Interact with it (e.g., touching, petting, etc.)	12 (2.00)
No reaction (e.g., any indifferent action like ignoring them)	155 (25.83)
**Respondents who observed dogs being killed in the community (respondents, *n* = 600)**	
Yes	96 (16.00)
No	504 (84.00)
**Dogs killed while wearing collars (respondents, *n* = 96)**	
Yes	15 (15.63)
No	67 (69.79)
Unsure	14 (14.58)
**Reasons for dogs with collars being killed (respondents, *n* = 15)**	
The dog bit someone	1 (6.67)
The dog was sick	12 (80.00)
The dog was being a nuisance in the community	1 (6.67)
The dog was hit by a car	1 (6.67)
**Reasons for dogs without collars being killed (respondents, *n* = 67)**	
The dog bit someone	15 (22.39)
The dog was sick	27 (40.30)
The dog was being a nuisance in the community	15 (22.39)
The dog was hit by a car	3 (4.48)
Unknown reason	7 (10.45)

**Table 3:** Dog owner participation during rabies vaccination events.

Characteristic	Frequency *n* (%)
**Number of dogs owned by respondents**	
Average	3
Min	1
Max	15
**Vaccination status of respondent’s dogs (respondents, *n* = 175)**	
All dogs vaccinated	122 (69.71)
Some dogs vaccinated	30 (17.14)
No dogs vaccinated	23 (13.14)
**Respondents who received a free rabies vaccination collar for their dog(s) (respondents, *n* = 152)**	
Yes	142 (93.42)
No	10 (6.58)
**Respondents who received dog collars after rabies vaccination**	
**Receiving a collar made respondents more likely to take their dog(s) for rabies vaccination (respondents, *n* = 142)**	
Yes	91 (64.08)
No	48 (33.80)
Unsure	3 (2.11)
**Respondents who believed that receiving a collar was an important sign that the dog had been vaccinated against rabies specifically (respondents, *n* = 142)**	
Yes	135 (95.07)
No	5 (3.52)
Unsure	2 (1.41)
**Collared status of respondent’s dogs (respondents, *n* = 142)**	
All dogs still wearing collars	47 (33.10)
Some dogs still wearing collars	81 (57.04)
No dogs wearing collars anymore	14 (9.86)
**Duration the collar was worn before it was lost (respondents, *n* = 95)**	
Less than a week	9 (9.47)
1 week	7 (7.37)
2 weeks	21 (22.11)
3 weeks	24 (25.26)
4 weeks	13 (13.68)
More than a month	21 (22.11)
**Who/What removed the collar (respondents, *n* = 95)**	
The dog’s owner (respondent)	16 (16.84)
The dog	14 (14.74)
Someone in the community	3 (3.16)
Unknown	62 (65.26)
**Reason for the owner removing the collar(s) (respondents, *n* = 16)**	
The dog(s) did not like wearing the collar(s)	2 (12.50)
To use the collar(s) for other purposes	0 (0.00)
The owner did not want the dog(s) to wear a collar(s)	4 (25.00)
The collar was hurting the dog	5 (31.25)
To stop the dog from getting stuck in the bush while hunting	4 (25.00)
The dog died	1 (6.25)
**Way the dog(s) removed the collar(s) (respondents, *n* = 14)**	
Collar lost while hunting	2 (14.29)
Collar lost while dogs were playing/fighting	8 (57.14)
Unknown	4 (28.57)
**Respondents who did not receive dog collars after rabies vaccination (respondents, *n* = 10)**	
**Reason why respondents did not receive a free collar for their dog(s)**	
No collar was offered by the vaccinator	9 (90.00)
The dog ran away before the collar could be put on it	1 (10.00)
**Respondents who did not have their dogs vaccinated against rabies (respondents, *n* = 19)**	
**Main reason respondent did not have their dog(s) vaccinated**	
The vaccine will make the dog(s) sick	0 (0.00)
The vaccine will change the behaviour of the dog(s)	0 (0.00)
The dog(s) was too young to be vaccinated	4 (21.05)
The respondent was unaware of the vaccination campaign	4 (21.05)
The respondent was not home during the vaccination campaign	5 (26.32)
No specific reason	6 (31.58)

## Data Availability

The survey data file is available from the Open Science Framework database (https://osf.io/56wkj/) (accessed on 27 July 2023).

## Supplementary Materials

The following supporting information can be downloaded at: https://www.mdpi.com/article/10.3390/tropicalmed8080421/s1, File S1: Questionnaire used during the KAP survey in Zanzibar. File S2: Overview of the vaccination date and survey date for each ward included in the study. File S3: Supplementary tables derived from the data collected in this study.
