# Supplementary material for: The Use of Dog Collars Offers Significant Benefits to Rabies Vaccination Campaigns: The Case of Zanzibar, Tanzania
Source: Trop Med Infect Dis. 2023 Aug 21;8(8):421. doi: 10.3390/tropicalmed8080421 (PMC10459019; doi:10.3390/tropicalmed8080421)
Supplement: Supplementary file 1 [file tropicalmed-08-00421-s001.zip › S1 File. Questionnaire used during the KAP survey in Zanzibar.pdf]

Questionnaire

Has the respondent agreed to take part in the survey voluntarily? (“Yes only” box)

Date of survey: ……………….…………….

Shehia name: ……………………………..

District: …………………………………….

Age of Respondent: ………………………

Sex of Respondent: ……………………….

S.no	Question	Responses	Skip Pattern
**Section A: General observation of vaccinated community dogs**			
A.1	Have you seen any dogs wearing collars around their neck in your community?	1 - Yes<br>2 - No	• If “1”, go to Question A.2.<br>• If “2”, go to Question B.1.
A.2	Please describe what the collars looked like that you saw on the dogs in your community?<br>*The surveyor will not give the answer and will not provide any guiding information to the respondent.*	1 – Any mention of plastic collars that are bright in color (i.e., “rabies vaccination collar”)<br>2 – Normal dog collars (i.e., NOT “rabies vaccination collar”)	• If “1”, go to Question A.3.<br>If “2”, go to Question B.1
A.3	Do you know what it means when a dog wears a plastic collar that is bright in color?<br>*The surveyor will not give the answer and will not provide any guiding information to the respondent.*	1 – The dog is vaccinated against rabies.<br>2 – The dog has been given an injection (rabies vaccination not mentioned specifically).<br>3 – The dog is healthy.<br>4 - The dog has a specific owner.	

		5 – Other (please specify).	
**Section B: General perception of community dogs**			
B.1	How do you feel if you encounter a dog with a bright colored collar in the community?	1 - I feel safer around dogs with those collars.<br>2 - No change in perception (neither safer nor more at-risk).<br>3 – I feel less safe around dogs with those collars.	
B.2	If you see a dog with a bright colored collar in the community, how would you typically react?	1 - Try to stay away from it.<br>2 - Try to chase it away (e.g., throwing something at it or trying to hit it with something).<br>3 – Interact with it (e.g., touching, petting, etc.).<br>4 – No reaction (e.g., any indifferent action like ignoring them)	
B.3	How do you feel if you encounter a dog without a bright colored collar in the community?	1 - I feel safer around dogs without collars.<br>2 - No change in perception (neither safer nor more at-risk).<br>3 – I feel less safe around dogs without collars.	
B.4	If you see a dog without a bright colored collar in the community, how would you typically react?	1 - Try to stay away from it.<br>2 - Try to chase it away (e.g., throwing something at it or	

		trying to hit it with something).<br>3 – Interact with it (e.g., touching, petting, etc.).<br>4 – No reaction (e.g., any indifferent action like ignoring them)	
B.5	Do you know of any dogs being killed in your community in the last 6 months?	1 - Yes<br>2 - No<br>3 - Don't know	• If "1", go to Question B.6.<br>• If "2" or "3", go to Question C.1.
B.6	Did any of the dogs that were killed have bright colored collars on?	1 - Yes<br>2 - No<br>3 - Don't know	
B.7	Do you know why the dogs were killed?<br>*The surveyor will not give the answer and will not provide any guiding information to the respondent.*	1 - They bit people.<br>2 - They were sick.<br>3 - They were being a nuisance in the community (e.g., killing chickens, etc.).<br>4 - Don't know<br>5 - Other (please specify)	
**Section C: Health seeking behavior**			
C.1	Have you been bitten by a dog with a bright colored collar in the last 4 weeks?	1 - Yes<br>2 - No	• If "1", go to Question C.2.<br>• If "2", go to Question D.1.
C.2	Why do you think the dog bit you?	1 - I tried to chase it away (e.g., throwing something at it or trying to hit it with something)<br>2 - I tried to interact with it (e.g., touching, petting, etc.)	

		3 - It just bit me for no reason.<br><br>4 - Other (please specify).	
C.3	Did you go for treatment at a healthcare facility after the bite?<br><br>*If the respondent answers "No" they will be advised to seek primary healthcare as soon as possible.*	1 - Yes<br><br>2 - No	• If "1", go to Question C.4.<br>• If "2", go to Question C.5.
C.4	Why did you decide to go for treatment?	1 - It was a severe bite wound and I needed a doctor/nurse to provide treatment.<br><br>2 - I was afraid of getting sick from the dog bite and wanted treatment.<br><br>3 - A community member told me to go.<br><br>4 - Other reason (please specify)	• Go to Question D.1.
C.5	Why did you decide <u>not</u> to go for treatment?	1 - Dogs with the bright collars are safe.<br><br>2 - Dogs with ANY collars are safe.<br><br>2 - The treatment is too expensive or inaccessible (facility too far away).<br><br>3 - It was not a severe bite, and I did not think treatment was needed.<br><br>4 - I used traditional remedies instead.	• Go to Question D.1

		5 - A community member told me not to go.	
**Section D: Rabies vaccination behavior**			
If necessary, the surveyor will inform the respondent that the "rabies vaccination collars" means that the dog has been vaccinated against rabies.			
D.1	Do you own any dogs?	1 - Yes<br>2 - No	• If "1", go to Question D.2.<br>• If "2", go to Question D.18.
D.2	If yes, how many dogs do you own?	Free answer	
D.3	Have your dog(s) been vaccinated against rabies <u>this year</u>?	1 - Yes, all the dogs.<br>2 - Yes, but only some of the dogs.<br>3 - No, none of the dogs.<br>4 - I don't know	• If "1" or "2", go to Question D.4.<br>• If "3", go to Question D.17.<br>• If "4", go to Question D.18.
D.4	Did you get a free collar for your dog(s) from the vaccinator?	1 - Yes<br>2 - No	• If "1", go to Question D.5.<br>• If "2", go to Question D.15.
D.5	Did getting a free collar for your dog(s) make you more likely to take your dog(s) for rabies vaccination?	1 – Yes<br>2 – No<br>3 – Don't know	
D6.	Is it important to you that the collar is a sign that the dog had been vaccinated against rabies specifically?	1 – Yes<br>2 – No<br>3 – Don't know	
D.7	How long ago did the dog(s) receive the collars?<br><br>*The surveyor will help the participant determine the timeframe.*	1 – Less than a week ago.<br>2 - 1 week ago<br>3 – 2 weeks ago<br>4 – 3 weeks ago<br>5 – 4 weeks ago<br>6 – more than a month ago	

D.8	Does your dog(s) still have the collar on?	1 - Yes, all the dogs.<br>2 - Yes, but only some of the dogs.<br>3 - No, none of the dogs.	• If "1", go to Question D.14.<br>• If "2" or 3, go to Question D.9.
D.9	How long did the dog wear the collar for?<br><br>*The surveyor will help the participant determine the timeframe. If multiple dogs have lost their collars, the average time must be selected.*	1 – Less than a week<br>2 - 1 week<br>3 – 2 weeks<br>4 – 3 weeks<br>5 – 4 weeks<br>6 – More than a month<br>7 – Don't know	
D.10	Who removed the collar?<br><br>*The surveyor will clearly specify that nobody will be identified as there will be no repercussions.*	1 - The owner<br>2 – The dog<br>3 – Someone in the community<br>4 - Unknown	• If "1", go to Question D.11.<br>• If "2", go to Question D.13.<br>• If "3" or "4", go to Question D.14.
D.11	Why did you remove the collar(s)?	1 - The dog(s) did not like wearing the collar(s).<br>2 - To use the collar(s) for other purposes.<br>3 – I did not want my dog(s) to wear a collar(s),<br>4 – The collar was hurting my dog.<br>5 – Other (Please specify)	
D.12	Was it easy to remove the collar?	1 – Yes<br>2 - No	• Go to question D.14
D.13	If possible, please explain how the dog removed the collar.	Free answer	• Go to question D.14

D.14	What can be done to improve the collar / make you more likely to keep the collar on the dog?	Free answer	• Go to question D.18
D.15	Why did you not get a free collar for the dog?	1 – The vaccinator did not offer me a collar for my dog(s).<br>2 – My dog(s) ran away before the collar could be put on.<br>3 – I did not want to put a collar on my dog(s).<br>4 – Other reason.	• If "1" or "2", go to Question D.18<br>• If "3" or "4", go to Question D.16
D.16	Why did you not want a free collar for your dog?	1 - The dog(s) will not like wearing the collar(s).<br>2 - I did not want my dog(s) to wear a collar(s).<br>3 – The collar was going to hurt my dog.<br>5 – Other (Please specify)	• Go to Question D.18.
D.17	What is the <u>main reason</u> for why you did <u>not</u> take your dog(s) for vaccination?	1 - I want my dog(s) to be healthy and the vaccine makes them sick.<br>2 – The vaccine changes the behavior of dogs (e.g., happy dogs become aggressive).<br>3 - Because I was told not to do it by someone in my community.<br>4 - I don't know	

		5 - Other (please specify)	
D.18	Have you seen anyone removing the bright colored collars from their dogs in the community?<br><br>*The surveyor will clearly specify that this is a "Yes/No" answer only. Nobody will be identified as there will be no repercussions.*	1 - Yes<br><br>2 – No	• If "1", go to Question D.20.<br>• If "2", Finish survey
D.19	Do you know why the collars were removed?	1 - The dog(s) did not like the collar.<br><br>2 - To use the collars for other purposes.<br><br>3 - The people did not want their dogs to wear collars.<br><br>4 - I don't know.	• Finish survey
