# Supplementary material for: The Use of Dog Collars Offers Significant Benefits to Rabies Vaccination Campaigns: The Case of Zanzibar, Tanzania
Source: Trop Med Infect Dis. 2023 Aug 21;8(8):421. doi: 10.3390/tropicalmed8080421 (PMC10459019; doi:10.3390/tropicalmed8080421)
Supplement: Supplementary file 1 [file tropicalmed-08-00421-s001.zip › S2 File. Overview of the vaccination date and survey date for each ward included in the study.pdf]

District	Shehia name	Required # of surveys	# of surveys undertaken	Date dogs vaccinated	Date survey undertaken	Days between events
North A	Chaani masingini	10	10	28-Nov-22	04-Feb-23	68
North A	Kigunda	10	10	08-Dec-22	08-Feb-23	62
North A	Kijini	10	10	06-Dec-22	06-Feb-23	62
North A	Kinyasini	10	10	29-Nov-22	06-Feb-23	69
North A	Matemwe	10	10	05-Dec-22	07-Feb-23	64
North A	Mkwajuni	10	10	01-Dec-22	09-Feb-23	70
North A	Mto wa pwani	10	10	02-Dec-22	09-Feb-23	69
North A	Pwani mchangani	10	10	13-Dec-22	07-Feb-23	56
North B	Donge Mtambile	10	10	05-Dec-22	09-Feb-23	66
North B	Kitope	10	10	30-Nov-22	08-Feb-23	70
North B	Kiwengwa	10	10	15-Dec-22	13-Feb-23	60
North B	Mafufuni	10	10	06-Dec-22	03-Feb-23	59
North B	Mkataleni	10	10	01-Dec-22	02-Feb-23	63
North B	Pangeni	10	10	02-Dec-22	13-Feb-23	73
North B	Zingwe Zingwe	10	10	12-Dec-22	07-Feb-23	57
Central	Bungi	15	15	01-Dec-22	09-Feb-23	70
Central	Charawe	15	16	29-Nov-22	13-Feb-23	76
Central	Dunga kiembeni	15	16	13-Dec-22	02-Feb-23	51
Central	Koani	15	15	15-Dec-22	06-Feb-23	53
Central	Mchangani	15	16	09-Dec-22	07-Feb-23	60
Central	Mgeni haji	15	15	12-Dec-22	05-Feb-23	55
Central	Miwani	15	15	14-Dec-22	14-Feb-23	62
Central	Ndijani mseweni	15	15	07-Dec-22	08-Feb-23	63
Central	Pagali	15	15	09-Dec-22	05-Feb-23	58
Central	Tindini	15	15	30-Nov-22	09-Feb-23	71

Central	Uroa	15	15	05-Dec-22	15-Feb-23	72
West A	Bububu	10	10	30-Nov-22	08-Feb-23	70
West A	Chuini	10	10	12-Dec-22	07-Feb-23	57
West A	Kianga	10	10	14-Dec-22	02-Feb-23	50
West A	Kizimbani	10	10	29-Nov-22	02-Feb-23	65
West A	Mfenesini	10	10	06-Dec-22	06-Feb-23	62
West A	Mtopepo	10	10	22-Dec-22	03-Feb-23	43
West A	Mwakaje	10	10	13-Dec-22	06-Feb-23	55
West A	Mwera	10	10	01-Dec-22	01-Feb-23	62
West B	Dimani	10	10	16-Dec-22	06-Feb-23	52
West B	Fumba	10	10	19-Dec-22	06-Feb-23	49
West B	Fuoni Kibondeni	10	10	04-Dec-22	07-Feb-23	65
West B	Fuoni Kijito Upele	10	10	30-Nov-22	03-Feb-23	65
West B	Kombeni	10	10	16-Dec-22	05-Feb-23	51
West B	Pangawe	10	10	30-Nov-22	03-Feb-23	65
West B	Shakani	10	10	15-Dec-22	05-Feb-23	52
West B	Tomondo	10	12	28-Nov-22	08-Feb-23	72
South	Dongwe	10	10	01-Dec-22	07-Feb-23	68
South	Kajengwa	10	11	02-Dec-22	02-Feb-23	62
South	Kibuteni	10	10	07-Dec-22	08-Feb-23	63
South	Muyuni B	10	10	13-Dec-22	06-Feb-23	55
South	Paje	10	10	02-Dec-22	02-Feb-23	62
South	Kitogani	10	10	06-Dec-22	05-Feb-23	61
Urban	Amani	5	6	29-Nov-22	03-Feb-23	66
Urban	Karakana	5	7	28-Nov-22	07-Feb-23	71
Urban	Kikwajuni Juu	5	6	06-Dec-22	08-Feb-23	64

Urban	Kilimani	5	9	08-Dec-22	04-Feb-23	58
Urban	Kiponda	5	5	07-Dec-22	05-Feb-23	60
Urban	Makadara	5	12	30-Nov-22	06-Feb-23	68
Urban	Mpendae	5	7	08-Dec-22	04-Feb-23	58
Urban	Rahaleo	5	7	05-Dec-22	06-Feb-23	63
