# Supplementary material for: The Use of Dog Collars Offers Significant Benefits to Rabies Vaccination Campaigns: The Case of Zanzibar, Tanzania
Source: Trop Med Infect Dis. 2023 Aug 21;8(8):421. doi: 10.3390/tropicalmed8080421 (PMC10459019; doi:10.3390/tropicalmed8080421)
Supplement: Supplementary file 1 [file tropicalmed-08-00421-s001.zip › S3 File. Supplementary tables derived from the data collected in this study.pdf]

Supplementary file 3.
Supplementary tables derived from the data collected in this study.

Table S1. Univariate and multivariable logistic regression analysis of the association between the socio-demographic variables and "feeling safer when encountering a dog with a collar".

Variable	Categories	Feeling safer when encountering a dog with a collar *		Total	OR (95% CI)	aOR (95% CI)
		Yes	No			
Sex	Female	71	80	151	Reference	Reference
	Male	270	179	449	1.6996 (1.1725 - 2.4636)	1.4640 (0,9771 - 2,1937)
Age	18 – 29	120	101	221	Reference	
	30 – 39	99	62	161	1.3440 (0.8890 - 2.0318)	
	40 – 49	59	41	100	1.2112 (0.7507 - 1.9542)	
	50 - 60	35	36	71	0.8183 (0.4791 - 1.3976)	
	>60	28	19	47	1.2404 (0.6541 - 2.3520)	
Setting	Urban	95	126	221	Reference	Reference
	Rural	246	133	379	2.4532 (1.7465- 3.4457)	2.8355 (1,9424 - 4,1393)
Dog ownership status	No	190	235	425	Reference	Reference
	Yes	151	24	175	7.7818 (4.8576 - 12.4664)	8.1235 (4.9857- 13.2363)

OR: Odds Ratio.
aOR: Adjusted Odds Ratio.
CI: Confidence interval.

*** Predictor variables retained in the multivariable logistic regression analysis: 'Dog ownership status' and 'Setting'**

Table S2. Univariate and multivariable logistic regression analysis of the association between the socio-demographic variables and "feeling less safe when encountering a dog without a collar".

Variable	Categories	Feeling less safe when encountering a dog without a collar *		Total	OR (95% CI)	aOR (95% CI)
		Yes	No			
**Sex**	Female	96	55	151	Reference	
	Male	302	147	449	1.1770 (0.8004 - 1.7309)	
Age	18 – 29	148	73	221	Reference	
	30 – 39	108	53	161	1.0051 (0.6525 - 1.5482)	
	40 – 49	63	37	100	0.8398 (0.5128 - 1.3755)	
	50 - 60	45	26	71	0.8537 (0.4885 - 1.4920)	
	>60	34	13	47	1.2900 (0.6419 - 2.5924)	
Setting	Urban	120	101	221	Reference	Reference
	Rural	278	101	379	2.3167 (1.6339 - 3.2847)	2.3564 (1.6458 - 3.3737)
Dog ownership status	No	254	171	425	Reference	Reference
	Yes	144	31	175	3.1273 (2.0266 - 4.8257)	3.1786 (2.0448 - 4.9410)

OR: Odds Ratio.
aOR: Adjusted Odds Ratio.
CI: Confidence interval.

*** Predictor variables retained in the multivariable logistic regression analysis: 'Sex' and 'Dog ownership status'**

Table S3. Health seeking behavior in response to dog bites.

Characteristic	Frequency *n* (%)
**Respondents bitten by dogs in 4 weeks leading up to survey (respondents, *n = 600*)**	
Yes	8 (1.33)
No	592 (98.67)
**Reason why the dog bit the respondent (respondents, *n* = *8*)**	
Tried to chase the dog away	1 (12.50)
Tried to interact with the dog	5 (62.50)
No obvious reason	2 (25.00)
**Respondents who sought treatment after exposure (respondents, *n* = *8*)**	
Yes	6 (75.00)
No	2 (25.00)
**Reason respondents sought treatment after exposure (respondents, *n* = *6*)**	
Bite wound that needed treatment	1 (16.67)
Rabies treatment specifically	4 (66.67)
Suggested by a community member	1 (16.67)
**Reason respondents did not seek treatment after exposure (respondents, *n* = 2)**	
Dogs with the bright collars are safe	0 (0.00)
Dogs with any collars are safe	0 (0.00)
Treatment too expensive	1 (50.00)
Traditional remedies were used	0 (0.00)
The exposure was not severe	1 (50.00)

Table S4. Univariate and multivariable logistic regression analysis of the association between the socio-demographic variables and "being more likely to take dogs for vaccination if collars are given".

Variable	Categories	Being more likely to take dogs for vaccination if collars are given*		Total	OR (95% CI)	aOR (95% CI)
		Yes	No			
Sex	Male	71	51	122	Reference	
	Female	20	0	20	56.9223 (3.3651 - 962.875)	
Age	18 – 29	39	26	65	Reference	
	30 – 39	24	18	42	0.8889 (0.4045 - 1.9532)	
	40 – 49	7	4	11	1.1667 (0.3101 - 4.3889)	
	50 - 60	14	2	16	4.6667 (0.9782 - 22.2638)	
	>60	7	1	8	4.6667 (0.5418 - 40.1947)	
Setting	Urban	37	3	40	Reference	Reference
	Rural	54	48	102	0.0912 (0.0264 – 0.3149)	0,1082 (0,0306 – 0,3823)

OR: Odds Ratio.
aOR: Adjusted Odds Ratio.
CI: Confidence interval.

*** Predictor variables retained in the multivariable logistic regression analysis: 'Sex'.**
